# Preliminary Evaluation of Probiotic Properties of *Lactobacillus* Strains Isolated from Sardinian Dairy Products

**DOI:** 10.1155/2014/286390

**Published:** 2014-06-26

**Authors:** Maria Barbara Pisano, Silvia Viale, Stefania Conti, Maria Elisabetta Fadda, Maura Deplano, Maria Paola Melis, Monica Deiana, Sofia Cosentino

**Affiliations:** ^1^Department of Public Health, Clinical and Molecular Medicine, University of Cagliari, Cittadella Universitaria, S.S. 554, Km 4,5, 09042 Monserrato, Italy; ^2^Department of Biomedical Sciences, Unit of Experimental Pathology, University of Cagliari, Cittadella Universitaria, S.S. 554, Km 4,5, 09042 Monserrato, Italy

## Abstract

Twenty-three *Lactobacillus* strains of dairy origin were evaluated for some functional properties relevant to their use as probiotics. A preliminary subtractive screening based on the abilities to inhibit the growth of microbial pathogens and hydrolyze conjugated bile salts was applied, and six strains were selected for further characterization including survival under gastrointestinal environmental conditions, adhesion to gut epithelial tissue, enzymatic activity, and some safety properties. All selected strains maintained elevated cell numbers under conditions simulating passage through the human gastrointestinal tract, well comparable to the values obtained for the probiotic strain *Lactobacillus rhamnosus* GG, and were able to adhere to Caco-2 cells to various extents (from 3 to 20%). All strains exhibited high aminopeptidase, and absent or very low proteolytic and strong *β*-galactosidase activities; none was found to be haemolytic or to produce biogenic amines and all were susceptible to tetracycline, chloramphenicol, erythromycin, ampicillin, and amoxicillin/clavulanic acid. Our results indicate that the *Lactobacillus* strains analyzed could be considered appropriate probiotic candidates, due to resistance to GIT simulated conditions, antimicrobial activity, adhesion to Caco-2 cell-line, and absence of undesirable properties. They could be used as adjunct cultures for contributing to the quality and health related functional properties of dairy products.

## 1. Introduction

Lactic acid bacteria (LAB) are widespread in nature and are one of the major microbial groups involved in the fermentation of different types of food. They represent the dominant microorganisms in milk and milk products where they play important roles during both manufacture and ripening. LAB possess a large number of metabolic properties that are responsible for the organoleptic characteristics of the final product as well as its preservation and microbial safety [1]. They are also known for their potential health and nutritional benefits and therefore are considered “probiotics” or “live microorganisms which upon ingestion in adequate amounts confer health benefits to the host” [2].

As a result of increasing awareness of the close interrelationship between health and diet, special attention is presently given to functional properties of LAB associated with traditional fermented products. These foods could represent alternative sources of novel probiotic candidates with physiological and functional properties for potential biotechnological use in the manufacturing of functional products in which the probiotic cultures are more active and protected from the gastrointestinal stress.

Among food ecosystem, cheese has some intrinsic chemical and physical characteristics (high pH, buffering capacity, solid consistency, high fat content, and high nutrient availability) that make it suitable for delivery of viable probiotic microorganisms into the human intestine [3]. Raw milk and traditional cheeses are rich environments with a varied and complex autochthonous microbiota composed of diverse group of microorganisms, including LAB, which contribute to the biopreservation and the development of organoleptic properties of the final product. LAB may play different roles in cheese-making; some species designated as starter LAB (SLAB) participate in the fermentation process, whereas some others indicated as nonstarter LAB (NSLAB) are implicated in the maturation process [4].

Among LAB,* Lactobacillus* strains have been extensively exploited for their probiotic properties [5] and are applied as adjunct cultures in various types of food products [6].

*Lactobacilli* are generally regarded as safe (GRAS) due to their long history of safe use and their presence in the intestinal microbiota of humans, and several species have received the qualified presumption of safety (QPS) status [7, 8].

Recently an increasing interest was paid to the possibility that strains belonging to species such as* Lactobacillus plantarum*,* L. paracasei*, and* L. brevis,* which constitute the majority of NSLAB found in most ripened cheese varieties, may play a role as health promoters, beyond their technological function [9].

To perform their probiotic action these bacteria must arrive at the intestinal tract alive. This requires their survival during food processing, product maturation, and shelf-life and, after consumption, their resistance to the acidic conditions of the stomach as well as to bile salts in the small intestine [10].

As a part of a larger study involving the selection and characterization of new probiotic candidates, 23 autochthonous* Lactobacillus* strains of dairy origin were investigated for some functional properties relevant to their use as probiotic cultures. Antagonism toward microbial pathogens, ability to deconjugate bile salts, survival in the gastrointestinal tract, adhesion capability to intestinal epithelial cells, enzymatic activities, and some safety features such as hemolytic activity, antimicrobial resistance, and production of biogenic amines were* in vitro* analyzed.

## 2. Materials and Methods

### 2.1. Bacterial Strains and Growth Conditions

A total of 23* Lactobacillus* strains (10* L. paracasei*, 9* L. plantarum*, and 4* L. brevis*) isolated from raw milk and artisanal ewes' cheeses were included in this study. The strains were identified on the basis of phenotypic tests and genetic analysis based on polymerase chain reaction amplification using species-specific primers derived from 16S rRNA sequences, as previously reported [11]. The strains were maintained at −20°C in MRS broth (Microbiol, Cagliari, Italy) with 15% (v/v) glycerol and propagated three times in MRS broth for activation prior to experimental use.* Listeria monocytogenes* ATCC 7644,* Escherichia coli* ATCC 35150,* Enterococcus faecalis* ATCC 29212,* Salmonella* Typhimurium ATCC 14028,* Staphylococcus aureus* ATCC 25923, and* Candida albicans* ATCC 10231 were used as indicators. All indicator strains were stored on nutrient broth (Microbiol) plus 20% (v/v) glycerol at −20°C. Before use, they were subcultured twice in appropriate medium.

### 2.2. Antimicrobial Activity

The strains were screened for antimicrobial compounds production against the indicator strains using an agar spot method [12]. Overnight cultures of* Lactobacilli* were spotted onto the surface of MRS agar (1.2% (w/v) agar—0.2% (w/v) glucose) plates, which were then incubated anaerobically for 24 h at 37°C. The indicator strains were inoculated into 7 mL of soft agar medium (nutrient broth containing 0.7% w/v agar) to a final concentration of approximately 10^7^ colony forming unit (cfu)/mL; then the soft media were poured on the plates. After 24 h of incubation at the optimal growth temperature and atmosphere for the indicator strains, inhibition halos were measured. The width of the clear zone (*R*) was calculated as follows: *R* = (*d*_Inhib_ − *d*_Spot_)/2, where *d*_Inhib_ is the diameter of the zone without pathogen growth and *d*_Spot_ is the diameter of the spot.

### 2.3. Bile Salt Hydrolase Activity

Bile salt hydrolase (BSH) activity was screened by spotting in duplicate 10 *μ*L of cultures grown overnight in MRS broth on the surface of MRS agar plates supplemented with 0.5% (w/v) sodium salt taurodeoxycholic acid (TDCA, Sigma, Milano, Italy) or 0.2% (w/v) glycodeoxycholic acid (GDCA, Sigma) and 0.37 g/L of CaCl_2_ [13]. Plates were incubated in anaerobic conditions at 37°C for 72 h. The presence of halos around colonies (in MRS-GDCA) or white opaque colonies (in MRS-TDCA) indicated BSH activity. MRS agar plates without supplementation were used as negative controls.* Enterococcus faecalis* ATCC 14433 was used as BSH-positive strain.

### 2.4. *In Vitro* Resistance to Gastrointestinal Conditions

Transit tolerance in the upper GIT was assessed using an* in vitro* model simulating gastric and pancreatic juices (simulated stomach-duodenum passage, SSDP), as reported by Vizoso Pinto et al. [14]. The strains were inoculated to a final concentration of approximately 2.5 × 10^8^ cfu/mL in 10 mL of simulated gastric juice (6.2 g/L NaCl, 2.2 g/L KCl, 0.22 g/L CaCl_2_, 1.2 g/L NaHCO_3_, 0.3% pepsin, and pH 3.0) and incubated at 37°C in a shaking water bath (Dubnoff 750, Asal, Milano, Italy) to simulate peristalsis. After 90 min, 17.5 mL of synthetic duodenum juice (6.4 g/L NaHCO_3_, 0.239 g/L KCl, 1.28 g/L NaCl, and 0.1% pancreatin), adjusted to pH 7.4 with 5 M HCl, and 4 mL of 10% (w/v) oxgall (Sigma) were added to the cell suspensions to simulate passage into the upper intestinal tract [15]. After 0, 90, and 180 min of incubation, the survival rate was determined by the plate method using MRS incubated anaerobically at 37°C for 48 h. The experiments were repeated twice and results are expressed as the mean log cfu/mL.

### 2.5. Adhesion Properties to Human Cell Line

The human colonic carcinoma cell line Caco-2 (ECACC, Salisbury, UK) was routinely cultured in Dulbecco's modified Eagle's minimal essential medium DMEM (Sigma), supplemented with 10% (w/v) foetal bovine serum (FBS, Sigma), 1% (w/v) nonessential amino acids solution (Sigma), and antibiotic solution (100 U/mL penicillin, 100 *μ*g/mL streptomycin). Cells were maintained in T-75 culture flasks at 37°C in a 5% CO_2_ atmosphere. For adhesion assay, the Caco-2 cells were seeded at a concentration of 10^5^ cells/well in 6-well tissue culture plates (Falcon) to obtain confluence and cultured for 20 days prior to use in adhesion assay. The cell culture medium was changed on alternate days and replaced by fresh DMEM supplemented with 2% (w/v) FBS and without antibiotic at least 1 h before the adhesion assay. A 1 mL aliquot of* Lactobacillus* suspension (10^8^ ufc/mL in phosphate buffered saline, PBS) was added to each well of the tissue culture plate and incubated at 37°C in 5% CO_2_ atmosphere for 3 h. Afterwards, the cells were washed three times with 1 mL of PBS in order to remove nonadherent bacteria and lysed by addition of Triton X 100 (0.05% solution) for 10 min; then appropriate dilutions were plated on MRS agar. Adhesion was expressed as the percentage of bacteria adhered to Caco-2 cells compared to the initial amount of bacteria.

### 2.6. Enzymatic Activities

Enzymatic activities of* Lactobacillus* strains were evaluated by using the API ZYM galleries (BioMérieux, Itay) as described by the manufacturer. The results were graded from 0 to 5 by comparing the colour developed within 5 min with the API-ZYM colour reaction chart. The results were expressed in nanomoles of hydrolysed substrate, from the intensity of the reactions obtained, in the range 0 (no activity) to 5 (40 or more nanomoles liberated), following the manufacturer's instructions.

### 2.7. Safety Assessment

The method of Bover-Cid and Holzapfel [16] was used to screen* Lactobacillus* strains for the production of biogenic amines. Briefly, the test strains were subcultured twice at 24 h intervals in MRS broth containing 1% of each precursor amino acid: tyrosine disodium salt, l-histidine monohydrochloride, l-ornithine monohydrochloride, and lysine monohydrochloride (Sigma), and 0.005% pyridoxal-5-phosphate (Sigma) as a codecarboxylase factor. All strains were then streaked in duplicate on decarboxylase medium plates each containing only one of the abovementioned amino acids and bromocresol purple as pH indicator and incubated for 4 days in anaerobic conditions at 37°C. Decarboxylase medium without amino acids was used as control. A colour change from brown to purple in the medium indicated an increase in pH and was considered a positive result.

Antibiotic susceptibilitytesting was carried out by disc diffusion method according to the Clinical and Laboratory Standards Institute (CLSI) guidelines [17] but Mueller-Hinton agar was replaced by MRS agar (Microbiol). The following antibiotics (Oxoid or BBL) were tested: ampicillin (AM; 10 *μ*g), amoxicillin/clavulanic acid (AmC; 30 *μ*g), vancomycin (VA; 30 *μ*g), teicoplanin (TEC; 30 *μ*g) (inhibitors of cell wall synthesis), tetracycline (TE; 30 *μ*g), streptomycin (S; 10 *μ*g), kanamycin (K; 30 *μ*g), gentamicin (GM; 10 *μ*g), chloramphenicol (C; 30 *μ*g), erythromycin (E; 15 *μ*g) (inhibitors of protein synthesis), ciprofloxacin (CIP; 5 *μ*g), and rifampicin (RA; 30 *μ*g) (inhibitors of nucleic acids). A suspension from fresh overnight cultures with a density of McFarland 0.5 in buffered saline was plated on MRS agar plates; then antibiotic discs were dispensed onto the plates. After incubation at 37°C for 24 h in anaerobiosis, the diameters of the bacterial free-zone were measured and results expressed in terms of resistance according to the interpretative criteria issued by the CLSI [18].

Haemolytic activity was determined by streaking the strains on Columbia Blood (Microbiol) agar plates supplemented with 5% defibrinated sheep blood after 48 h of incubation at 37°C. The haemolytic reaction was recorded by observation of a clear zone of hydrolysis around the colonies (*β*-haemolysis), a partial hydrolysis and greenish zone (*α*-haemolysis), or no reaction (*γ*-haemolysis).

### 2.8. Statistical Analysis

Statistical analysis of data was carried out using GraphPad Prism Statistics software package version 3.00 (GraphPad Prism Software Inc., San Diego, CA, USA). One-way ANOVA followed by Bonferroni test was used to determine significant differences of viability of the* Lactobacillus *strains during SSDP treatment and with respect to the adhesion ability. Data were analysed at the significance level of *P* < 0.05.

## 3. Results and Discussion

A preliminary subtractive screening based on the abilities to inhibit the growth of microbial pathogens and hydrolyse conjugated bile salts was applied to the strains. As shown in Table 1, most strains were able to inhibit the growth of* L. monocytogenes*,* S. aureus*, and* E. coli* O157:H7 with clear inhibition zones of more than 6 mm in agar-spot plates. A lower activity was detected against* E. faecalis,* while one strain showed an inhibition zone of 4 mm against* C. albicans *ATCC 10231. Such broad antagonistic activity of* Lactobacillus* towards different pathogens has been reported [19, 20]; however, 27 strains of* L. plantarum* isolated from cheese showed no activity against selected indicator pathogens [21].

LAB are known to produce many different substances with antimicrobial activity, including the major metabolic end products such as organic acids, hydrogen peroxide, ethanol, and bacteriocins [22]. When the strains with the best inhibitory activity in the agar spot-test were further tested using the well-diffusion assay, to investigate the presence of bacteriocin-like compounds, no inhibitory activity was observed (data not shown), presumably indicating that the production of organic acids was responsible for the observed antimicrobial effect. The inhibitory effect of hydrogen peroxide was excluded due to incubation in anaerobic conditions.

BSH activity is a relevant property for probiotic strains to survive the toxicity of conjugated bile salts in the duodenum [23]. In our study, while all strains were able to grow in the presence of conjugated bile salts after 72 h of incubation, only five (*L. plantarum* 11/20966, 4/16868, 19/20711 and* L. paracasei *62LP39, 1A6 M) demonstrated also the ability to hydrolyze both sodium glycodeoxycholate (GDCA) and sodium taurodeoxycholate (TDCA), and four were able to deconjugate only GDCA, as indicated by the BSH test on agar plates. Some authors have shown that in* Lactobacilli* the resistance to bile salts toxicity is not related to hydrolase activity [21, 24–26]. Other mechanisms, alternative to BSH, could be involved in counteract bile damage, as suggested by Noriega et al. [27].

BSH activity is frequently observed in specific groups of bacteria like* Lactobacillus*,* Bifidobacterium*, and* Enterococcus* isolated from the gastrointestinal tract (GIT): it has been reported for* L. plantarum* [14, 28] but not for* L. paracasei* strains [26, 29]. Presently its role is controversial since it has been reported to act either positively in lowering of serum cholesterol [30] or negatively in increasing the level of undesirable deconjugated bile salts [31]. However, the bacterial genera most frequently used as probiotics (*Bifidobacteria* and* Lactobacilli*) are not capable of dehydroxylating deconjugated bile salts [32, 33] and so the majority of the breakdown products may be precipitated and excreted with feces [34]. On the other hand, BSH activity by a probiotic bacterium may be desirable since it increases the intestinal survival and persistence of producing strains, which in turn increases the overall beneficial effects associated with the strain [35].

Among the BSH positive strains, five showing the ability to ferment the trisaccharide raffinose, known for its prebiotic activity, and one with a high antagonistic activity against* C. albicans* ATCC 10231 were selected for further functional characterization including* in vitro* tests to assess their survival under gastrointestinal environmental conditions and adhesion to gut epithelial tissue and to determine the enzymatic activity and some safety properties.

The survival responses of the strains, after 90 and 180 min of exposure to different gastrointestinal conditions, are shown in Table 2. The probiotic strain* L. rhamnosus* GG was included in the study for comparison purpose. All strains retained high viability during simulated gastric juice (pH 3.0) transit for 90 min, and no significant reduction (*P* > 0.05) was found for any of the strains after exposure to artificial duodenum juice (pH 8.0). The strains* L. paracasei* 62LP39 and* L. plantarum* 11/20966 and 4/16868 presented the best survival rates values with more than 98% surviving cells.

Exposure to gastric and intestinal fluids is the main environmental stress that decreases viability of ingested probiotics [36]. All selected strains maintained elevated cell numbers under conditions simulating passage through the human gastrointestinal tract, well comparable to the values obtained for the probiotic strain* L. rhamnosus* GG. The survival rates were above 90% after 3 h despite the high bile concentration (more than 1%) used in the test. These results are consistent with those previously reported for other potentially probiotic strains belonging to* L. paracasei* [26] and* L. plantarum* [21] and correlate well with those obtained for BSH activity.

**Figure 1:**
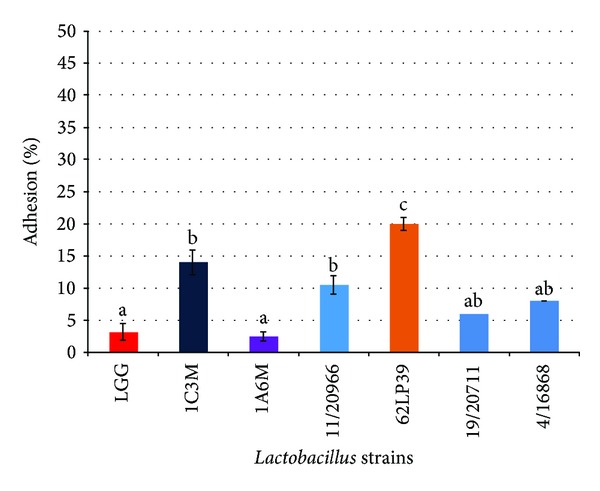
Adhesion ability to Caco-2 human colon cell lines of dairy* Lactobacillus* strain (mean ± E.S. of three independent experiments). Mean values with different superscript letters were significantly different at *P* < 0.05.

The adhesion ability of* Lactobacillus* strains is reported in Figure 1. All strains were able to adhere to Caco-2 cells to various extents (ranging from 3 to 20%), confirming that adhesion is a strain-specific property. Three strains showed significantly higher adherence to Caco-2 cells than* L. rhamnosus* GG (*P* < 0.05).* L. paracasei* 62LP39 was the most adhesive strain since approximately 20% of the added bacteria were bound to Caco-2 cells, followed by* L. brevis* 1C3 M (14%) and* L. plantarum* 11/20966 (10.5%). A lower adhesion rate was observed for the strain* L. paracasei* 1A6 M (3%). The percentage of adhesion of* L. rhamnosus* GG, used as positive control, was similar to those obtained with the same strain in other studies [37, 38] but lower than the values found by Xu et al. [39].* L. plantarum* strains have been shown to adhere more efficiently to Caco-2 cells than* L. paracasei* or* L. brevis* [29, 38], although Toumola and Salminen [37] obtained an adhesion percentage at the level of 6.7%. Other authors observed that* L. paracasei* strains were effective in reducing adhesion of Salmonella to Caco-2 cells in competitive assays, in spite of their low adherence ability [40–42].

Several* in vitro* studies suggested that probiotic adhesion may interfere with the adherence of pathogens, exerting a barrier against pathogen colonization through competitive exclusion mechanisms [39, 40].

Enzymatic characterization of the selected strains was carried out in order to evaluate their potential for using as adjunct cultures in the manufacturing of probiotic cheese and is reported in Table 3. All strains exhibited high aminopeptidase activity (leucine and valine arylamidase) while esterase and lipase activities were generally moderate to low. The proteolytic and N-acetyl-*β*-glucosaminidase activities were absent or very low. All strains exhibited strong *β*-galactosidase and moderate-to-high *α*-galactosidase activities, which are responsible for the hydrolysis of lactose and raffinose, respectively. None of the strains showed *β*-glucuronidase or *α*-mannosidase activity.

Overall, the enzymatic profiles of our strains are similar to those reported by other authors particularly for the species* L. plantarum *[42–44]. Mathara et al. [43] could not detect any *β*-galactosidase in 14* L. paracasei* isolates and Herreros et al. [42] found a certain degree of activity in two strains of* L. brevis*. Strains with low proteinase, high peptidase, and low esterase-lipase activities may be useful in reducing bitterness and improving body and texture defects [43, 44]. *β*-galactosidase activity is an important feature for strains to be used as probiotics, since it may be useful in improving lactose tolerance in the gut [45]. The lack of *β*-glucuronidase activity is an important trait, as well, since its negative role has been suggested in increasing the risk of carcinogenesis [46].

In order for a strain to be used as probiotic culture, it should be evaluated for the presence of virulence determinants to determine what potential risks might be involved in its use. Biogenic amines are produced by LAB during the process of fermentation of foods and beverages by amino acid decarboxylation. Bover-Cid and Holzapfel [16] suggested that the capability to produce biogenic amines in a synthetic medium might be strain dependent rather than being related to specific species. In our screening, none of the strains tested was found to decarboxylate lysine, histidine, ornithine, or tyrosine (data not shown), in agreement with other findings [29, 47].

The* Lactobacillus* strains were assayed for their resistance to 12 antibiotics using a disc diffusion method on MRS agar plates under anaerobic conditions, and the results are reported in Table 4. Within the group of antimicrobial agents that inhibit the cell wall synthesis, all strains were resistant to vancomycin and teicoplanin and susceptible to ampicillin and amoxicillin/clavulanic acid. All strains were susceptible to tetracycline, chloramphenicol, and erythromycin, moderately susceptible to gentamycin, and resistant to streptomycin and kanamycin. As for the antibiotics that inhibit the nucleic acids synthesis, all strains were resistant to ciprofloxacin and susceptible to rifampicin.

Our results are in substantial agreement with those reported for* L. plantarum* and* L. paracasei* strains [26, 29], although other authors observed a certain variability in strains' reaction to tetracycline [19, 48].

The resistance observed against some antibiotics tested suggests that our strains would not be affected by therapies using these antibiotics and might help maintain the natural balance of intestinal microflora during antibiotic treatments [49]. Resistance to some antibiotics such as aminoglycosides, quinolones, and glycopeptides appears to be intrinsic for* Lactobacilli* [50, 51]. In particular, vancomycin resistance is well documented in* Lactobacilli* and it has been attributed to the synthesis of modified cell wall peptidoglycan precursors that end in a depsipeptide d-alanine-d-lactate instead of the dipeptide d-alanine-d-alanine, the target for vancomycin activity [52]. Moreover, Klein et al. [50] showed that the glycopeptide resistance in* Lactobacillus* strains is not of the transmissible type. The strains tested in our work do not seem to represent a source for transfer of genes encoding resistance, since they were phenotypically susceptible to tetracycline, erythromycin, and chloramphenicol, but the absence of the genes needs to be confirmed genotypically. Comunian et al. [48] recently found that tetracycline and erythromycin resistance is not usually detected in strains originating from cheeses produced in geographical areas (such as Sardinia island) where no systematic use of antibiotics as growth promoters was carried out over the years in animal husbandry.

Finally, none of the strains was found to be haemolytic (data not shown), in agreement with previous studies [53].

## 4. Conclusions

In conclusion, our results indicate that some* Lactobacillus* strains (particularly* L. paracasei* 62LP39,* L. plantarum* 11/20966 and 4/16868, and* L. brevis* 1C3 M) could be considered appropriate probiotic candidates, due to resistance to GIT simulated conditions, antimicrobial properties, adhesion to human intestinal epithelial cell-line (Caco-2), and absence of undesirable properties. They could be used as adjunct cultures for contributing to the quality and health related functional properties of dairy products; however, additional studies are required to confirm* in vivo* this findings as well as to assess the strains stability to manufacturing processes.

## Figures and Tables

**Table 1:** Antimicrobial activity against the microbial pathogens tested, bile salt hydrolase (BSH) activity, and raffinose fermentation of selected potential probiotic *Lactobacillus* strains isolated from Sardinian dairy products.

Strains	Target strains						Hydrolysis of		
	*S. aureus* ATCC 25923	*L. monocytogenes* ATCC 7644	*E. coli* ATCC 35150	*E. faecalis * ATCC 29212	*S. Typhimurium* ATCC 14028	*C. albicans * ATCC 10231	TDCA	GDCA	Raffinose∗
*L. paracasei* 62LP39	7.5	9	6.5	1.5	>10	2	+	+	+
*L. paracasei *8/18710	5	9	7.5	2	>10	1.5	−	−	−
*L. paracasei *1A6M	6	7.5	6	2	>10	1	+	+	+
*L. paracasei *3A1M	3	6	5	1	7.5	0	−	−	−
*L. paracasei *5/22019	6.5	8.5	5	0.5	7.5	0	−	+	−
*L. paracasei *2C6M	6	5	6	0.5	7.5	0	−	−	−
*L. paracasei* 60LP37	7.5	9	9	1	>10	0.5	−	−	−
*L. paracasei *2A11	6.5	9	5	0.5	>10	0	−	−	−
*L. paracasei *6B1M	5	7.5	6	1	>10	0.5	−	−	−
*L. paracasei *2B1M	5	9	6	1.5	>10	1	−	−	−
*L. plantarum *11/20966	7	9	7.5	1	>10	2.5	+	+	+
*L. plantarum *19/20711	7.5	9	7.5	2	>10	2	+	+	+
*L. plantarum *4/16868	10	10	9	2.5	>10	2	+	+	+
*L. plantarum *8C1M	5	5	6	1.5	>10	0	−	+	−
*L. plantarum *9FS15	6	1.5	5	1.5	>10	1.5	−	−	−
*L. plantarum *28SP1	4.5	2.5	3	1	>10	1.5	−	−	−
*L. plantarum *2AFS11	6.5	9	5	1	>10	0.5	−	−	−
*L. plantarum *5FS12	6	5	6	0.5	7.5	0	−	−	−
*L. plantarum *12LP13	5	7.5	6	1	>10	0	−	−	−
*L. brevis *1C3M	6.5	7.5	6	0.5	>10	4	−	+	−
*L. brevis *9/11B	5	9	5	0.5	>10	0.5	−	+	−
*L. brevis *21FS1B	5	7	5	1	>10	0.5	−	−	−
*L. brevis *9A3M	4	5	5	0	>10	0.5	−	−	−

∗Determined by API-50 CHL galleries (bioMérieux).

**Table 2:** Resistance of *Lactobacillus* strains to simulated stomach duodenum-passage (SSDP). The values are reported as log⁡⁡cfu/mL (mean ± E.S.).

Species	Initial mean count	Survival after 90 min at pH 3.0	Survival after 180 min of which 90 min in 5% oxgall
Strain			
*L. paracasei *			
62LP39	8.33 ± 0.03	8.67 ± 0.11	8.66 ± 0.84
1A6M	8.49 ± 0.07	8.08 ± 0.02	7.47 ± 1.39
*L. plantarum *			
11/20966	8.66 ± 0.15	9.70 ± 0.91	8.62 ± 0.17
19/207111	8.58 ± 0.13	8.58 ± 0.3	7.76 ± 0.35
4/16868	8.21 ± 0.03	8.06 ± 0.2	8.47 ± 0.2
*L. brevis *			
1C3M	8.59 ± 0.23	8.75 ± 0.05	8.39 ± 0.14
*L. rhamnosus* GG	8.59 ± 0.02	9.03 ± 0.18	8.23 ± 0.59

**Table 3:** Enzymatic profiles of selected dairy *Lactobacillus* strains assayed by the API-ZYM system.

Enzyme tested	Strains					
	* L. paracasei *		*L. plantarum *			* L. brevis *
	62LP39	1A6M	11/20966	19/20711	4/116868	1C3M
Alkaline phosphatase	20	10	10	5	10	5
Esterase (C4)	10	5	5	0	5	20
Esterase lipase (C8)	10	10	5	10	10	10
Lipase (C14)	10	20	10	10	20	20
Leucine arylamidase	>40	>40	>40	30	>40	>40
Valine arylamidase	>40	>40	30	10	30	>40
Cystine arylamidase	20	20	10	0	20	20
Trypsin	5	5	0	0	5	0
*α*-Chymotrypsin	0	0	5	5	5	5
Acid phosphatase	20	30	20	20	20	30
Napthol-AS-BI-phosphohydrolase	30	20	10	10	10	10
*α*-Galactosidase	20	10	10	0	0	30
*β*-Galactosidase	>40	30	>40	10	20	>40
*β*-Glucuronidase	0	0	0	0	0	0
*α*-Glucosidase	30	30	20	0	20	30
*β*-Glucosidase	30	10	30	20	30	30
N-Acetyl-glucosaminidase	5	0	0	5	0	0
*α*-Mannosidase	0	0	0	0	0	0
*α*-Fucosidase	0	5	0	0	0	0

Enzyme activity is expressed as the approximate nanomoles of hydrolysed substrate after 4 h of incubation at 37°C.

**Table 4:** Antibiotic susceptibility of selected *Lactobacillus *strains.

Species	Strain	Antibiotic tested∗											
		AM10	AmC30	VA30	TEC30	TE30	S10	K30	GM10	C30	E15	CIP30	RA30
*L. paracasei *	62LP39	S	S	R	R	S	R	R	MS	S	S	R	S
	1A6M	S	S	R	R	S	R	R	MS	S	S	R	S

*L. plantarum *	11/20966	S	S	R	R	S	R	R	MS	S	S	R	S
	19/20711	S	S	R	R	S	R	R	MS	S	S	R	S
	4/16868	S	S	R	R	S	R	R	MS	S	S	R	S

*L. brevis *	1C3M	S	S	R	R	S	R	R	MS	S	S	R	S

∗Antibiotics: (AM) ampicillin; (AmC) amoxicillin/clavulanic acid; (VA) vancomycin; (TEC) teicoplanin; (TE) tetracycline; (S) streptomycin; (K) kanamycin; (GM) gentamicin; (C) chloramphenicol; (E) erythromycin; (Cip) ciprofloxacin; (RA) rifampicin. R: resistant; S: sensitive; MS: moderately susceptible.
